# HO-1 Is Essential for Tetrahydroxystilbene Glucoside Mediated Mitochondrial Biogenesis and Anti-Inflammation Process in LPS-Treated RAW264.7 Macrophages

**DOI:** 10.1155/2017/1818575

**Published:** 2017-04-03

**Authors:** Weihua Yu, Xiaodi Zhang, Hao Wu, Qingbiao Zhou, Zhao Wang, Rui Liu, Jiangzheng Liu, Xin Wang, Chunxu Hai

**Affiliations:** Department of Toxicology, The Ministry of Education Key Lab of Hazard Assessment and Control in Special Operational Environment, Shanxi Key Lab of Free Radical Biology and Medicine, School of Public Health, The Fourth Military Medical University, Xi'an 710032, China

## Abstract

2,3,5,4′-Tetrahydroxystilbene-2-O-*β*-D-glucoside (TSG), an important monomer extracted from Polygonum multiflorum, can prevent a number of inflammation associated chronic diseases. However, the mechanism involved in TSG inducing anti-inflammatory role remains unclear. As an inducible antioxidant enzyme, Heme oxygenase-1 (HO-1), is crucial for protecting the mammalian cells against adverse stimuli. Here, we found that the TSG treatment strongly induces the expression of HO-1 in an NRF2-depended manner. Meanwhile, TSG increased the mitochondrial mass through upregulation of the mitochondrial biogenesis activators (PGC-1*α*, NRF1, and TFAM) as well as the mitochondrial complex IV. Furthermore, TSG attenuated Lipopolysaccharide (LPS) mediated RAW264.7 cells activation and secretion of proinflammatory cytokines, including interleukin-6 (IL-6) and tumor necrosis factor-*α* (TNF-*α*). Zinc Protoporphyrin (ZnPP), a selective inhibitor of HO-1 activity, was able to attenuate TSG mediated mitochondrial biogenesis and anti-inflammatory process. Finally, we observed that LPS induced obvious mtDNA depletion and ATP deficiency, which indicated a severe damage of mitochondria. TSG restored the LPS induced mitochondrial dysfunction via activation of the mitochondrial biogenesis. ZnPP treatment markedly reversed the inhibitory effects of TSG on mitochondrial damage and oxidative stress in LPS stimulated macrophages. Taken together, these findings suggest that TSG enhances mitochondrial biogenesis and function mainly via activation the HO-1. TSG can be developed as a potential drug for treatment of inflammatory diseases.

## 1. Introduction

Inflammation is a vital contributor to a series of chronic diseases, such as neurodegenerative diseases, heart diseases, alcoholic hepatitis, diabetes, tumors, and senility [1–4]. The inflammatory process is involved with activation of several immune cells including macrophages, lymphocytes, and neutrophils. In particular, macrophage plays an important role in the inflammation procession by inducing overexpression of a series of inflammatory cytokines and mediators [2, 5]. It has been reported that the extract of Polygonum multiflorum has various pharmacological activities, including antioxidant activities, anti-inflammation, delay senility, and improvement of memory ability [6–11]. As a major bioactive component of Polygonum multiflorum, TSG has been confirmed to inhibit the inflammatory processions in vivo and in vitro [12]. However, the mechanism of TSG associated anti-inflammation effects is still unclear.

Mitochondria play a key role not only in maintaining normal cellular homeostasis but also in the procession of pathological conditions [13]. Excessive ROS and inflammation can cause mitochondrial damage, which in turn induces acute and chronic pathological responses, including multiorgan failure, neurodegeneration, and cardiovascular disease [1, 14, 15]. Recent researches indicate that the promotion of mitochondrial biogenesis and quality will enhance cell survival and repairmen in various disease states. In mammalian cells, mitochondrial biogenesis is activated by both physiological and pathological procedures, such as cell division, exercise, calorie restriction, oxidative stress, and inflammation [16–18]. Mitochondrial biogenesis is regulated by a complex network of factors, including peroxisome proliferator-activated receptor gamma coactivator 1-alpha (PGC-1*α*), nuclear factor-erythroid derived 2-related factor 1 (NRF1), and mitochondrial transcription factor A (TFAM) [19]. These transcription factors and coactivators regulate mitochondrial proteins synthesis and mitochondrial DNA (mtDNA) replication. Hence, mitochondrial biogenesis promotes cellular recovery from damage through influencing the population and function of mitochondria [20, 21]. In this study, we explored the effect of TSG on mitochondrial biogenesis and inflammation in LPS induced RAW264.7 macrophages.

ROS plays a central role during the macrophages activation, which may cause excessive inflammation, tissue damage, and sepsis [22]. However, most cell types have developed defensive mechanisms to scavenge excessively produced ROS. As a member of the intracellular phase II enzyme family, Heme oxygenase-1 (HO-1) is crucial for maintaining cellular redox homeostasis. Recent research demonstrated that overexpression of HO-1 markedly suppressed TNF-*α* inducing airway inflammation by inhibition of oxidative stress [23]. Moreover, animal models with HO-1 deficiency were susceptible to severe inflammation [24, 25], while the role of HO-1 in TSG inducing anti-inflammatory property has not been evaluated yet.

In the present study, we verified that TSG suppressed the proinflammatory cytokines production in LPS-treated RAW264.7 cells. TSG strongly stimulated HO-1 expression via NRF2 pathway in LPS stimulated RAW264.7 cells. Moreover, TSG promoted mitochondrial biogenesis via activation of a series of transcription factors, including PGC-1*α*, NRF1, and TFAM. Intervention with HO-1 inhibitor significantly attenuated the TSG mediated mitochondrial biogenesis and anti-inflammation procession. These findings suggested that TSG enhanced mitochondrial biogenesis mainly via inducing the HO-1 expression. TSG was able to promote mitochondrial quality and used as therapeutics for the treatment of diseases involving inflammation and mitochondrial dysfunction.

## 2. Materials and Methods

### 2.1. Reagents

2,3,5,4′-Tetrahydroxystilbene-2-O-*β*-D-glucoside was purchased from Chengdu MUST Biotechnology company (Chengdu, China) with 99.25% purity. Lipopolysaccharide, Dexamethasone, Zinc Protoporphyrin, DAPI, and ATP Assay kit were purchased from the Sigma-Aldrich (St. Louis, USA). Brusatol was obtained from the TAUTO Biotechnology (Shanghai, China). The molecular probes including Mito-tracker red CMXRos, Mito-tracker green, and MitoSOX™ were obtained from Invitrogen Corporation (Carlsbad, CA, USA). Mouse specific ELISA kit for IL-6 and TNF-*α* level was purchased from CUSABIO Biotechnology Corp. (Wuhan, China). Mitochondrial complex I activity detecting kit was bought from Nanjing Jiancheng Bioengineering Institute (Nanjing, China). Real-Time PCR primers for GAPDH, beta2-microglobulin, cytochrome b, PGC-1*α*, NRF1, and TFAM, IL-6, TNF-*α*, SOD2, SOD1, NQO-1, CAT, GPX-1, HO-1, and TNF-*α* were synthesized by AuGCT Biotechnology (Beijing, China). Real-Time PCR one-step cDNA synthesis kit, TIANamp Genomic DNA Kit, and Real-Time PCR amplification kit were purchased from TIANGEN Biotechnology (Beijing, China). COX2, IL-6, TNF-*α*, and *β*-actin antibodies were obtained from Santa Cruz Biotechnology (Santa Cruz, USA). Mitochondrial complex IV antibody, Annexin V-FITC/PI Apoptosis Detection Kit, and WST-1 Cell Proliferation and Cytotoxicity Assay Kit were bought from Beyotime Biotechnology (Shanghai, China). HO-1 and NRF2 antibodies and Goat anti-Rabbit IgG-(H+L)-HRP were purchased from Abcam (Cambridge, MA).

### 2.2. Cell Cultures and Treatment

Murine macrophage cell line RAW264.7 was bought from the American Type Culture Collection (ATCC, Manassas, VA). Cells were cultured in high glucose DMEM supplemented with 10% fetal bovine serum (Gibco BRL, Rockville, MD, USA). RAW264.7 cells were planted in either 6-well plates or 24-well plates and then incubated at 37°C in a humidified 5% CO_2_ atmosphere. After being pretreated with TSG (0–120 *μ*M) and Dex (100 nM/mL) for 6 hours, the macrophages were exposed to 1 *μ*g/mL LPS for 12 hours. Meanwhile, ZnPP (20 *μ*M) or Brusatol (50 *μ*M) were also used alone with TSG treatment. At the end, cells were harvested for the further experiments.

### 2.3. Detection of Cell Viability

RAW264.7 cells were exposed to various concentrations of TSG (0–480 *μ*M) for 6 hours. After treatment, cell viability was analyzed by using WST-1 Cell Proliferation and Cytotoxicity Assay Kit. Following incubation with WST-1 for 2 hours, the optical density was measured at 450 nm with a microplate reader (Olympus, Japan).

### 2.4. Apoptosis Assessment

RAW264.7 cells were exposed to various concentrations of TSG (0,120, 240, and 480 *μ*M) for 6 hours. Cell apoptosis rate was assessed with an Annexin V-FITC/PI Apoptosis Detection Kit. After incubation for 30 min at room temperature, RAW264.7 cells (10^4^ counts) were collected by flow cytometer (BD Accuri C6, USA).

### 2.5. Measurement of Proinflammatory Cytokines

RAW264.7 cells (2 × 10^5^ per well) were seeded in 24-well plates. After treatment, the culture supernatants were collected to detect the concentrations of TNF-*α* and IL-6 with the specific ELISA kits according to the manufacturer's instructions. The optical density of the samples was obtained at 540 nm with a microplate reader. The standard curve of proinflammatory cytokines was calibrated by using the professional soft “Curve Expert 1.3.” Finally, the TNF-*α* and IL-6 concentrations in culture supernatants were analyzed.

### 2.6. Mitochondrial Mass Measurement

To detect the changes of mitochondrial mass, RAW264.7 cells were stained with specific mitochondrial indicator probes, including Mito-tracker Red CMXRos and Mito-tracker green. After treatment, cells were incubated with Mito-tracker red CMXRos (500 nM) for 30 min at 37°C. Cells were analyzed by using a confocal laser scanning microscopy (Olympus, Japan). The excitation and emission wavelengths were set at 510 and 590 nm, respectively. Meanwhile, RAW264.7 cells were also stained with Mito-tracker green (500 nM) for 30 min at 37°C in the dark. After washing with PBS (pH = 7.4) for three times, cells were harvested and analyzed by a flow cytometer (BD Accuri C6). The fluorescence intensity of Mito-tracker green was analyzed by using the Image-Pro plus 6 software.

### 2.7. mtDNA Content Analysis

Total DNA was extracted with a TIANamp Genomic DNA Kit according to the manual protocol. Quantitative Real-Time PCR was performed to evaluate the mtDNA content with cytochrome b and beta2-microglobulin that served as probes specific for mtDNA and nuclear DNA. The primers were synthesized by AuGCT Biotechnology, and the sequences were as follows: mouse cytochrome b: forward primer 5′-TTTGGGTCCCTTCTAGGAGTC-3′, reverse primer 5′-CCGACATGAAGGAATAAGCAA-3′; murine beta2-microglobulin: forward primer 5′- ATGGGAAGCCGAACATACTG-3′, reverse primer 5′- CAGTCTCAGTGGGGGTGAAT-3′. Then, Real-Time PCR was conducted using a SuperReal PreMix Plus (SYBR green) detecting kit as the manufacturer's protocol description. The relative gene expression was analyzed according to the CFX Manager 2.1 software (Bio-Red, USA). Finally, mtDNA content was indicated by cytochrome b expression, which was normalized by beta2-microglobulin.

### 2.8. ATP Detection

It is well known that Adenosine 5′-triphosphate (ATP) acts as the major energy currency in living organisms. Here, luciferase's ATP determination kit was used to detect ATP level in RAW264.7 cells. The research is based on the fact that ATP is absolutely required when luciferase is producing light. After treatment, RAW264.7 cells were homogenized with the lysis buffer and centrifuged at 12000 ×g for 10 minutes. 100 *μ*l samples were mixed with 100 *μ*l reacting buffer. Luminescence of the standards and the samples were captured at 560 nm using a luminometer. Meanwhile, the amount of ATP was calculated based on the standard curve. ATP level was expressed as *μ*mol/g protein after it was normalized to the protein content in each sample.

### 2.9. Mitochondrial Complex I Activity

The mitochondrial complex I activity is indicated by the procession of oxidation NADH to NAD+, which results in increasing the absorbance at 340 nm. After treatment, RAW264.7 cells were homogenized with the lysis buffer for 30 minutes and then centrifuged at 12000 ×g for 20 minutes. Each experimental reagent was added to the plate according to the instructions and incubated for 3 min at 30°C in dark. Finally, 100 *μ*l of assay solution was added to each well and OD_340_ was measured at approximated one-minute intervals for 30 minutes. Mitochondrial complex I activity was expressed as (mOD/min)/*μ*g protein after the results were normalized to the protein content.

### 2.10. Mitochondrial ROS Detection

Mitochondrial ROS level was measured by staining with MitoSOX Red. It was a novel fluorescent probe, which was known for selective indication of superoxide anion in mitochondria of living cells. RAW264.7 cells were stained with 200 *μ*l MitoSOX (5 mM/L) for 30 min at 37°C. After washing, cells were collected by flow cytometry, and the fluorescence intensity of MitoSOX Red was analyzed with the Image-Pro plus 6 software.

### 2.11. Real-Time PCR Analysis

Briefly, the total RNA in RAW264.7 cells was obtained through one-step TRIzol extraction method. The complementary DNA was transformed by using the commercial kits according to the manufacturer's protocol (TIANGEN). Quantitative Real-Time PCR was carried out by using the CFX96 Real-Time PCR Detection System (Bio-Rad). The primers were synthesized by AuGCT Biotechnology, and the sequences were shown in Table 1. Real-Time PCR was conducted using a 20 *μ*l reaction system as described in SuperReal PreMix Plus (SYBR green). The relative gene expression was analyzed with the CFX Manager 2.1 software and then normalized to the amount of GAPDH.

### 2.12. Western Blot Analysis

Briefly, RAW264.7 cells were lysed on ice for 30 min in RIPA Lysis Buffer. The lysates were centrifuged at 20,000 ×g for 20 min. The protein concentrations in supernatants were detected with a BCA Protein Assay Kit (Pierce). Then, equal amounts of protein extractions (30 mg) were separated by using SDS-polyacrylamide gel electrophoresis (10–15%) and then blotted to polyvinyl difluoride membranes. The membrane was treated with primary antibodies about 12 hours at 4°C. After washing, the blots were incubated with corresponding anti-IgG which was conjugated with horseradish peroxidase for 1 h at 37°C. At the end, the membranes were visualized with enhanced chemiluminescence after exposure to X-ray. The protein abundance was quantified by using a Bio-Rad Imaging System.

### 2.13. Statistical Analysis

All of the data in this article were analyzed with SPSS statistical software. Statistical analysis was carried out by using the one-way analysis of variance (ANOVA) and Student's *t*-test. The results were expressed as the mean ± SEM (standard error of the mean). *P* < 0.05 was considered significantly different between two groups.

## 3. Results

### 3.1. TSG Attenuated LPS Inducing Macrophages Activation and Proinflammatory Cytokines Production

**Figure 1:**
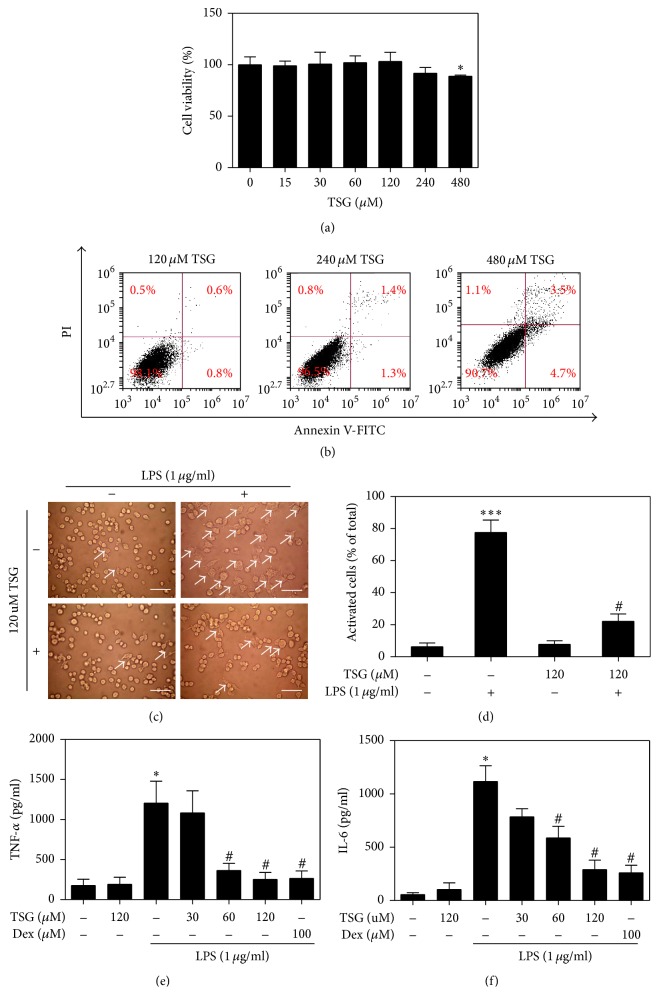
TSG reduced RAW264.7 cells activation and proinflammatory cytokines release induced by LPS. RAW264.7 cells were exposed to various concentrations of TSG (0–480 *μ*M) for 6 hours. (a) Cell viability was analyzed by WST assay. (b) Annexin V-PI method was used to detect the cell death rate. (c-d) RAW264.7 cells were exposed to 1 *μ*g/mL LPS for 12 hours with or without TSG (120 *μ*M) pretreatment for 6 hours. Then, the morphological change was captured under a microscope. Bar = 50 *μ*m (×30). The percentage of activated cells in total cell population was calculated. (e-f) In the presence and absence of TSG (0, 30, 60, and 120 *μ*M) or Dex (100 *μ*M) for 6 hours, RAW264.7 macrophages were stimulated with 1 *μ*g/mL LPS for 12 hours. The concentration of TNF-*α* and IL-6 production in the culture medium were assessed by specific ELISA kits. All data were expressed as the means ± SEM of three independent experiments. ^*∗*^*P* < 0.05 and ^*∗∗∗*^*P* < 0.001 versus control; ^#^*P* < 0.05 versus LPS treatment group.

Firstly, the effect of TSG on the cell viability was assessed in RAW264.7 macrophages. Treatment with 30–240 *μ*M TSG did not show any effect on cell viability, whereas there was an obvious reduction of cell viability at concentration of 480 *μ*M (Figure 1(a)). Meanwhile, the cell death rate has no significant difference after RAW264.7 cells exposure to TSG below 240 *μ*M (Figure 1(b)). Then, we attempted to examine whether TSG was able to suppress the LPS stimulated activation and inflammation in RAW264.7 macrophages. It was known that macrophages exhibited a spreading and elongated morphology with pseudopodium-like protrusions after activation [26]. We defined the small round shaped cells as resting cells and the enlarged ones with protrusions as activated cells (Figure 1(c)). After being treated with TSG and LPS, the morphological changes of cells were examined in RAW264.7 cells. Compared with the control group, the percentage of activated cells was obviously increased after stimulation with LPS for 12 hours. However, pretreatment with TSG notably blocked LPS mediating macrophages activation (Figure 1(d)).

The proinflammatory cytokines, such as TNF-*α*, IL-1*β*, and IL-6, are crucial for the procession of inflammation and recruitment of immune cells. Enzyme-linked immunosorbent assay (ELISA) was used to detect the content of inflammatory cytokines in cell culture medium. The results revealed that TSG (30–120 *μ*M) led to a concentration-dependent suppression of TNF-*α* and IL-6 production induced by LPS (Figures 1(e) and 1(f)). When macrophages were pretreated with 120 *μ*M of TSG for 6 hours, the inhibition rates of LPS induced TNF-*α* and IL-6 protein secretion were, respectively, about 78.9% and 77.8%, approximated to Dex cotreatment group. These results indicated that TSG mitigated the macrophages activation and inflammation in LPS-treated RAW264.7 cells.

### 3.2. TSG Promoted HO-1 Gene and Protein Expression in an NRF2 Dependent Way

**Figure 2:**
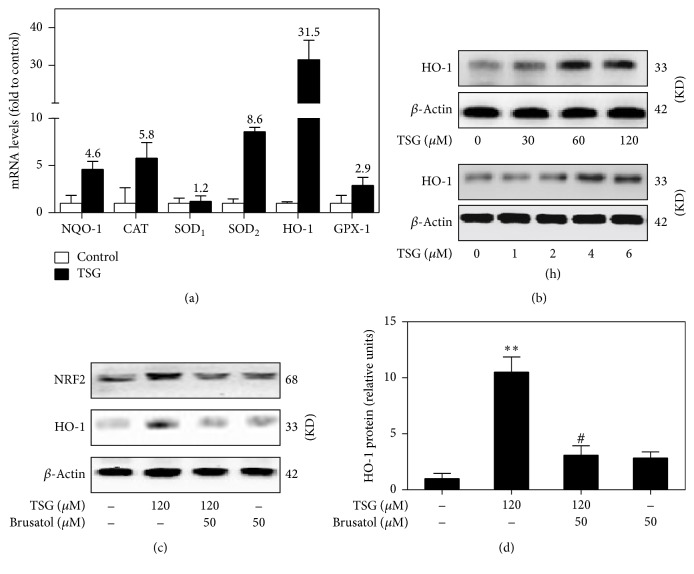
TSG stimulated HO-1 gene and protein expression via an NRF2 dependent pathway. (a) After RAW264.7 cells were stimulated with TSG (120 *μ*M) for 6 hour, the relative mRNA levels of HO-1, CAT, SOD_1_, SOD_2_, NQO-1, and GPX-1 were evaluated by Real-Time PCR. (b) RAW264.7 cells were treated with various concentrations (0, 30, 60, and 120 *μ*M) of TSG for indicated times (0, 1, 2, 4, and 6 h), and then HO-1 protein level was detected by western blot analysis. (c) RAW264.7 cells were exposed to TSG (120 *μ*M) for 6 hours in the presence and absence of Brusatol (50 *μ*M). Western blot analysis assessed NRF2 and HO-1 protein expression in cell lysates. (d) The relative HO-1 protein level was analyzed by densitometry. Data were expressed as the mean ± SEM of triplicate independent experiments (*n* = 3). ^*∗∗*^*P* < 0.01 versus control group; ^#^*P* < 0.05 versus TSG treatment group.

It has reported that NRF2 related antioxidant pathway was crucial for TSG mediating cell protective role in vivo and in vitro [27, 28]. Here, we detected the effects of TSG on NRF2-ARE driven antioxidant genes expression; the mRNA levels of HO-1, SOD_2_, SOD_1_, CAT, NQO-1, and GPX-1 were determined in the presence and absence of TSG. After RAW264.7 cells treatment with TSG (120 *μ*M) for 6 hours, the mRNA levels of NQO-1, CAT, SOD_1_, SOD_2_, HO-1, and GPX-1 increased 4.6-, 5.8-, 1.2-, 8.6-, 31.5-, and 2.9-fold, respectively (Figure 2(a)). As a member of the intracellular phase II enzyme family, HO-1 is thought to be crucial for maintaining cellular redox homeostasis. As shown in Figure 2(b), HO-1 protein sustained a low level in cells without stimulation, while it increased in a time- and dose-dependent manner after treatment with 30–120 *μ*M of TSG. To investigate whether TSG activated HO-1 via NRF2 pathway, Brusatol was used to downregulate NRF2 signaling. Compared with untreated macrophages, TSG induced a strong increase of NRF2 protein expression. However, Brusatol attenuated the TSG inducing NRF2 and HO-1 protein expression in RAW264.7 macrophages (Figures 2(c) and 2(d)). Therefore, we concluded that TSG promoted HO-1 gene and protein expression via NRF2 pathway.

### 3.3. HO-1 Was Essential for TSG Mediated Anti-Inflammatory Effect in LPS-Activated Macrophages

**Figure 3:**
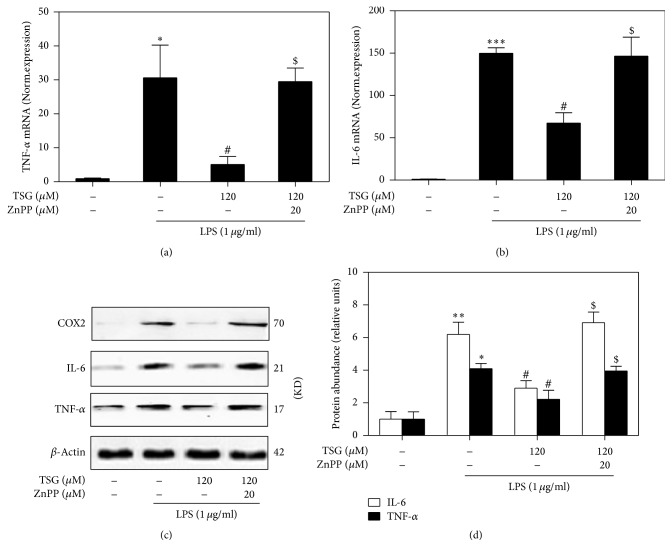
Effects of HO-1 on TSG mediated anti-inflammatory effect in LPS-activated macrophages. RAW264.7 cells were pretreated with TSG (120 *μ*M) for 6 h with or without 20 *μ*M of ZnPP and then stimulated with LPS (1 *μ*g/mL) for 12 h. (a-b) Relative mRNA expression of TNF-*α* and IL-6 was determined by Real-Time PCR. (c) Western blot analysis was performed to assess the expression of COX-2, IL-6, and TNF-*α* in whole cell lysates. (d) The relative IL-6 and TNF-*α* protein level was analyzed by densitometry. Data were expressed as the mean ± SEM of triplicate independent experiments (*n* = 3). ^*∗*^*P* < 0.05, ^*∗∗*^*P* < 0.01, and ^*∗∗∗*^*P* < 0.001 versus control group; ^#^*P* < 0.05 versus TSG treatment group; ^$^*P* < 0.05 versus LPS + TSG group.

Protoporphyrin IX zinc (ZnPP) was known as a specific inhibitor of HO-1 activity. Here, it was used to explore the role of HO-1 in TSG mediated anti-inflammatory activity. Real-Time PCR results showed that LPS induced TNF-*α* (Figure 3(a)) and IL-6 (Figure 3(b)) mRNA expressions increase about 30- and 150-fold, respectively. Pretreatment with TSG obviously attenuated the gene expression of these proinflammatory cytokines, while ZnPP intervention reversed these inhibition effects of TSG. Meanwhile, western blot results showed that TSG blocked the LPS induced protein upregulation of COX2, TNF-*α*, and IL-6. However, these processions could be attenuated by ZnPP treatment (Figures 3(c) and 3(d)). In sum, this further verified that TSG inducing anti-inflammatory activity was associated with activation of HO-1.

### 3.4. TSG Promoted the Mitochondrial Mass through Inducing the Transcription Factors Involved in Mitochondrial Biogenesis

**Figure 4:**
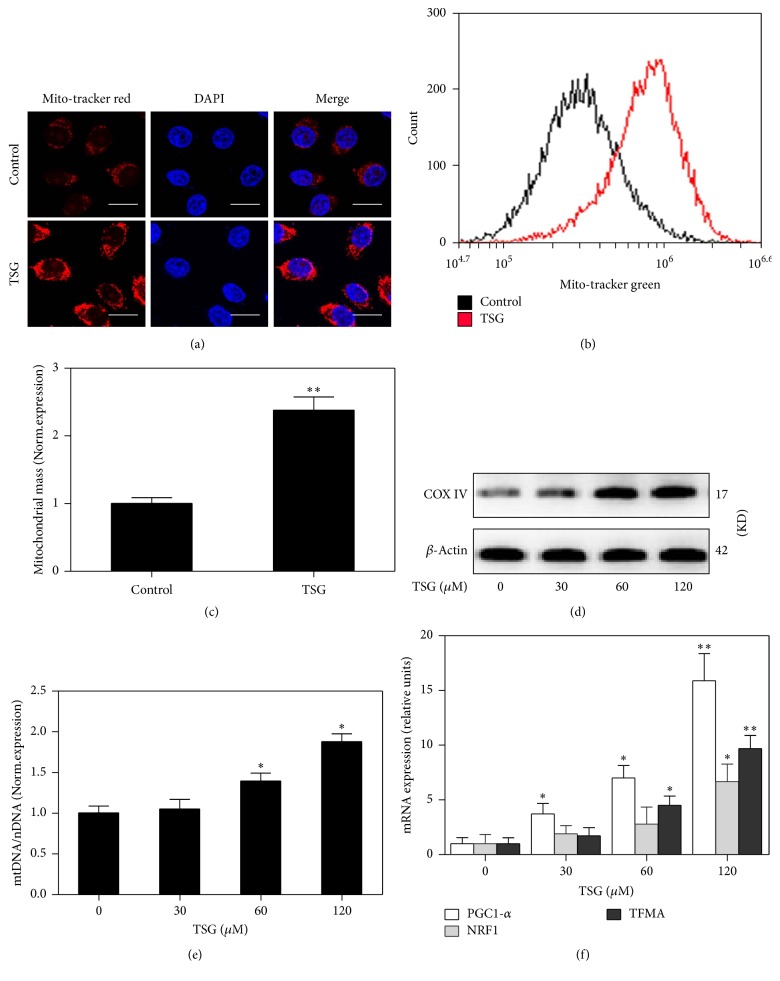
TSG promoted the mitochondrial biogenesis in RAW264.7 macrophages. RAW264.7 cells were treated with TSG (0, 30, 60, and 120 *μ*M) for 6 h. (a) Mito-tracker red CMX-Ros (red) and DAPI (blue) were used to stain the mitochondria mass and cell nucleus. Fluorescence images were captured by a confocal microscopy. Bar = 10 *μ*m (×240). (b) Mitochondrial mass was also assessed by flow-cytometry method with Mito-tracker green staining. (c) The Mito-tracker green fluorescence was assessed by using Image-Pro plus 6 software, and mean fluorescence was calculated. (d) Western blot analysis was performed to assess the expression of mitochondrial complex IV in whole cell lysates. (e) Quantitative PCR was performed to evaluate the mtDNA content. Relative ratios of mtDNA and nDNA contents were analyzed. (f) The mRNA expressions of PGC-1, NRF1, and TFAM were detected by Real-Time PCR methods. All data were expressed as means ± SEM of triplicate independent experiments. ^*∗*^*P* < 0.05 and ^*∗∗*^*P* < 0.01 versus control group.

Next, we detected the effect of TSG on the mitochondrial biogenesis in RAW264.7 cells. As a specific probe to stain mitochondria, Mito-tracker red (green) was used to indicate mitochondria population in living cells. As shown in Figure 4(a), TSG treatment induced a significant increasing of mitochondrial mass, which was marked by the enhancement of red fluorescence staining in macrophages. Similarly, the above results were also verified by flow cytometry analysis after RAW264.7 cells were stained with Mito-tracker green (Figures 4(b) and 4(c)). It has been reported that the increase in mtDNA copy number was an important index of mitochondrial biogenesis [29]. Therefore, Quantitative PCR was performed to evaluate the mtDNA content with beta2-microglobulin and cytochrome b serving as probes specific for nuclear DNA and mtDNA. After being treated with TSG, the mtDNA copy number obviously increased relative to control group (Figure 4(d)). Furthermore, TSG also stimulated the protein expression of mitochondrial complex IV in a dose-dependent way (Figure 4(e)). It was well known that mitochondrial biogenesis was regulated by a series of transcription factors and coactivators. Treatment of RAW264.7 cells with TSG (120 *μ*M) significantly augmented the mRNA expression of mitochondrial biogenesis related factors, including PGC-1*α*, NRF1, and TFAM (Figure 4(f)). These indicated that TSG promoted the mitochondrial mass through inducing the gene expression of mitochondrial biogenesis associated transcription factors.

### 3.5. Induction of Mitochondrial Biogenesis by TSG Was Associated with HO-1 Activation

**Figure 5:**
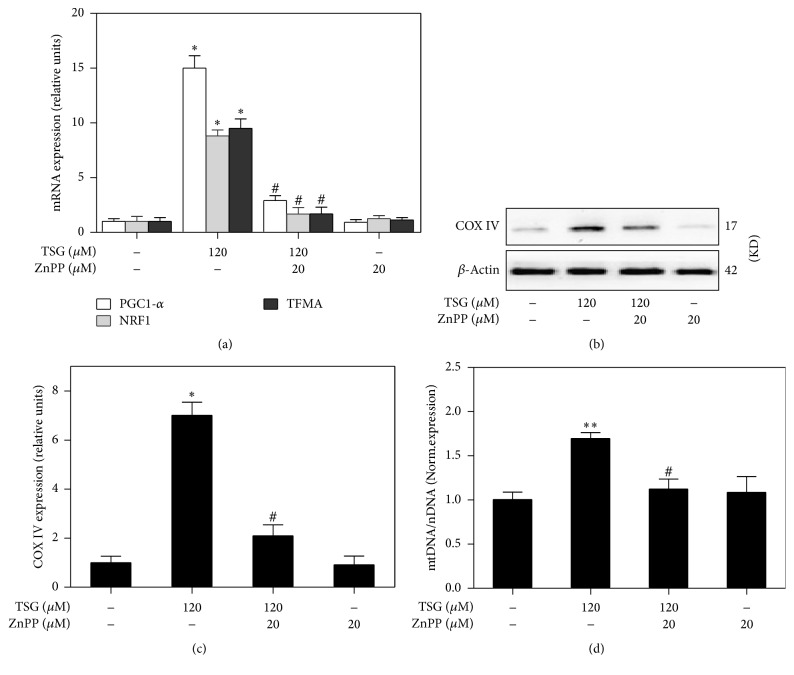
The mitochondrial biogenesis induced by TSG was regulated by activation of HO-1. RAW264.7 cells were treated with TSG (120 *μ*M) for 6 hours in the presence and absence of ZnPP (20 *μ*M). (a) After treatment, mRNA expressions of PGC-1, NRF1, and TFAM were detected by Real-Time PCR. (b) Western blot analysis was used to assess the expression of mitochondrial complex IV in whole cell lysates. (c) The relative COX-IV protein level was analyzed by densitometry. (d) Quantitative PCR was performed to evaluate the mtDNA content with beta2-microglobulin and cytochrome b served as probes specific for nuclear DNA and mtDNA. Relative ratios of mtDNA and nDNA contents were analyzed. All measurements were expressed as the mean ± SEM of triplicate independent experiments (*n* = 3). ^*∗*^*P* < 0.05 and ^*∗∗*^*P* < 0.01 versus control group; ^#^*P* < 0.05 versus TSG treatment group.

According to our previous study, TSG was able to promote HO-1 expression as well as mitochondrial biogenesis. It was known that CO induced by HO-1 might activate mitochondrial biogenesis [29]. Therefore, we wondered whether the activation of mitochondrial biogenesis by TSG involved the activation of the HO-1 in RAW264.7 cells. As a specific inhibitor of HO-1 activity, Protoporphyrin IX zinc (ZnPP) was used alone with TSG in RAW264.7 cells. As shown in Figure 5(a), TSG (120 *μ*M) obviously induced mRNA expression of PGC-1*α*, NRF1, and TFAM. Treatment of macrophages with ZnPP reversed TSG mediated gene expression of the abovementioned mitochondrial biogenesis related factors. Consistent with these findings, TSG also induced increase of COX-IV protein, while this effect was able to be antagonized by ZnPP (Figures 5(b) and 5(c)). Furthermore, the induction of mtDNA levels by TSG also was inhibited by treatment with ZnPP (Figure 5(d)). These results indicated that TSG was able to promote the mitochondrial biogenesis through activation of HO-1.

### 3.6. TSG Restored the Mitochondrial Function in LPS-Treated RAW264.7 Cells through Induction of HO-1

**Figure 6:**
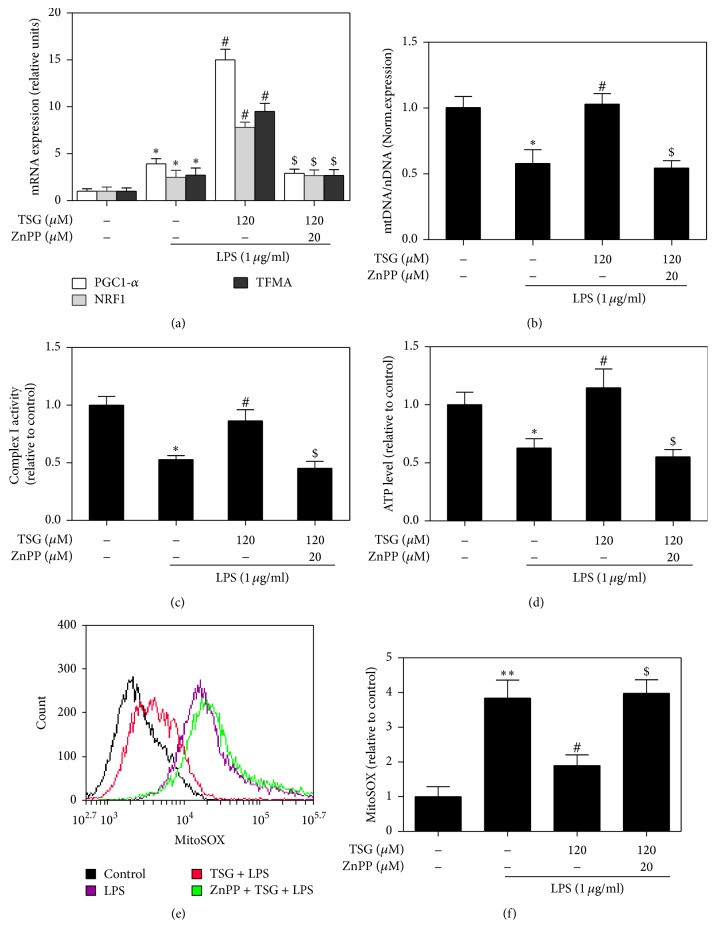
TSG restored mitochondrial function in LPS-treated RAW264.7 cells via an HO-1 dependent fashion. RAW264.7 cells were pretreated with TSG (120 *μ*M) for 6 h with or without 20 *μ*M of ZnPP and then stimulated with LPS (1 *μ*g/mL) for 12 h. (a) After treatment, the mRNA expressions of PGC-1, NRF1, and TFAM were detected by Real-Time PCR. (b) Quantitative PCR was performed to evaluate the mtDNA content. Relative ratios of mtDNA and nDNA contents were analyzed. (c) The mitochondrial complex I activity was detected by following the oxidation of NADH with a specific detecting kit. (d) The cellule ATP content was analyzed with a luciferase assay kit. (e) The superoxide anion content in RAW264.7 cells was detected by flow-cytometry method after staining with MitoSOX. (f) The MitoSOX fluorescence was analyzed by using Image-Pro plus 6 software. Data were expressed as the mean ± SEM of triplicate independent experiments (*n* = 3). ^*∗*^*P* < 0.05 and ^*∗∗*^*P* < 0.01 versus control group; ^#^*P* < 0.05 versus TSG group; ^$^*P* < 0.05 versus LPS + TSG group.

Finally, we examined the effects of TSG on the mitochondrial biogenesis and function in LPS-treated RAW264.7 cells. As shown in Figure 6(a), LPS increased the mRNA expression of PGC-1*α*, NRF1, and TFAM compared with untreated group. TSG pretreatment induced a further augmentation of these mitochondrial biogenesis transcription factors. It has been reported that there are serious oxidative stress and mitochondrial damage after macrophages exposure to stress stimuli, including LPS and TNF-*α* [30]. After treatment with LPS, we observed an obvious diminution in the level of mtDNA, while TSG pretreatment suppressed the decline of mtDNA content (Figure 6(b)). Likewise, TSG promoted the mitochondrial complex I activity and ATP content in LPS stimulated RAW264.7 macrophages (Figures 6(c) and 6(d)). However, ZnPP antagonized the protective role of TSG in LPS induced mtDNA depletion, ATP deficiency, and mitochondrial complex I activity reduction. Superoxide anion was known as the primary form of ROS existing in mitochondria. It was generated as a byproduct in the procession of oxidative phosphorylation. To evaluate the effect of TSG on mitochondria oxidative stress, RAW264.7 cells were detected by staining with MitoSOX (Figure 6(e)). The results showed that TSG was able to alleviated LPS-mediated superoxide anion production. However, inhibition of HO-1 activity with ZnPP obviously attenuated TSG inducing scavenge of mitochondria ROS (Figure 6(f)). These results indicated that TSG protected RAW264.7 cells against the mitochondrial damage induced by LPS through induction of HO-1.

## 4. Discussion

As a redox sensitive inducible protein, HO-1 is an important gene to protect cells against oxidative stress, inflammation, and apoptotic cell death [31]. Accumulating studies indicated that both mice and human deficiency in HO-1 expression have a phenotype of increased inflammatory state [24]. As an active component extracted from Polygonum multiflorum, TSG showed obvious protection effects in a series of diseases. NRF2 is a key regulator for cellular redox homeostasis and acts as a primary defense against oxidative stress. Here, our research demonstrated that TSG activated the NRF2-ARE driven antioxidant genes, including HO-1, SOD_2_, CAT, NQO-1, and GPX-1. Particularly, the relative HO-1 mRNA increased about 32-fold after RAW264.7 cells treatment with TSG for 6 hours. Inhibition of NRF2 with Brusatol was able to abolish TSG mediating HO-1 overexpression. These verified that TSG induced HO-1 expression through activation NRF2 pathway.

Meanwhile, we found that TSG was able to enhance the expression of PGC-1*α*, which acted as a master regulator of the transcriptional network involving mitochondrial biogenesis. PGC-1*α* activated NRF1 and TFAM, which could regulate the nuclear genes encoding proteins associated with mitochondrial respiratory chain and mtDNA replication [13, 19]. COX-I and COX-IV are important members of the mitochondrial respiratory chain. Its protein expression and enzyme activity determined the capacity of ATP synthesis and electrons transfer [32]. In the current experiment, we observed an obvious increase of COX-IV protein as well as the COX-I activity in TSG treated RAW264.7 cells. Mitochondrial quality control is important to protect cells against the adverse stress. This procession involves a strict regulation about the mitochondrial number, size, distribution, and phenotype. Here, we verified that TSG induced obviously increasing of the mitochondrial mass, which was detected by Mito-tracker red (green). These results indicated that TSG stimulated mitochondrial biogenesis and had a potential to improve the mitochondria quality.

It has been reported that HO-1 might play a crucial role during the procession of mitochondrial biogenesis [29, 33]. In this study, we found that TSG was able to induce a substantial increase of HO-1 expression as well as the mitochondrial biogenesis. As an inhibitor of HO-1, ZnPP blocked TSG inducing expression of mitochondrial biogenesis related transcription factors, including PGC-1*α*, NRF1, and TFAM. Meanwhile, TSG mediated increase of COX-IV protein and mtDNA content were also reduced after exposure to ZnPP. Hence, we concluded that TSG induced mitochondrial biogenesis depended on HO-1 activation. Meanwhile, the drugs that could strongly induce HO-1 single might be useful for diseases associated with mitochondrial dysfunction.

Macrophage activation is crucial for the development of multiple diseases via releasing plenty of ROS and inflammatory mediators [34–36]. In the present study, we clearly demonstrated that TSG attenuated LPS-mediated macrophages activation and inflammatory procession. LPS caused an obvious elevation of the inflammatory cytokines, including TNF-*α* and IL-6, while TSG exhibited a well anti-inflammatory effect in LPS stimulated macrophages. Interestingly, TSG inducing inhibition effect on TNF-*α* and IL-6 secretion was approximated to Dex, which was known as a positive drug for inflammation. Meanwhile, LPS induced increased HO-1 expression and mitochondrial biogenesis, which might be associated with the cellular self-defense against the inflammation induced mitochondria damage. Moreover, TSG further enhanced these processions through induction of more abundance of HO-1. Treatment with ZnPP attenuated TSG mediated anti-inflammation effects through reducing the HO-1 activity. Therefore, HO-1 is essential for TSG mediated mitochondrial biogenesis and anti-inflammation process in LPS-treated RAW264.7 macrophages.

Organisms often exhibited serious oxidative stress and mitochondrial damage during systemic inflammatory conditions, such as severe injury and acute infections [37, 38]. These processions could be blocked by enhancing the cellule anti-inflammatory and antioxidant defenses. Recent reports showed that mitochondrial biogenesis might play an important role during the cell defenses against the adverse stimuli [30, 39]. Here, we observed that LPS induced decline of the mitochondrial DNA copy number. Meanwhile, the mitochondrial complex I activity and the ATP content were also decreased after RAW264.7 cells exposure to LPS. ATP is generated during cellular respiration procession in mitochondria. It is a key indicator to show the quality and function of the mitochondria. Energy exhaustion and the mitochondrial complex I activity reduction indicated that LPS mediated severe mitochondrial dysfunction. However, TSG enhanced the mitochondrial biogenesis through inducing transcription factors, including PGC-1*α*, NRF1, and TFAM. Compared with LPS stimulated cells, TSG pretreatment obviously promoted the mitochondrial function, exhibited as the increase of mtDNA content, mitochondrial complex I activity, and ATP synthesis. It was well known that the superoxide anion played a central role during mitochondrial damage [40, 41]. We demonstrated that TSG could alleviate the level of superoxide anion induced by LPS treatment. Furthermore, ZnPP antagonized the protective effects of TSG with respect to the mitochondrial biogenesis and function through inhibited HO-1 activity. Hence, TSG restored the mitochondrial function in LPS-treated RAW264.7 cells through induction of HO-1.

Taken together, these results showed that TSG was able to suppress LPS stimulated macrophages activation and inflammation. As a redox sensitive inducible protein, HO-1 played a crucial role in the progress of TSG mediated mitochondrial biogenesis and function restoration. Strategies aimed at inducing HO-1 and mitochondrial biogenesis might be useful for treatment of sepsis and other diseases involving with mitochondrial dysfunction.

## Figures and Tables

**Table 1:** 

Primer	Sequences
PGC-1*α*	Forward, 5′-GTGTTCTGGTACCCAAGGCA-3′
	Reversed, 5′-GGAGACTGGGCCGTTTAGTC-3′
HO-1	Forward, 5′- GAAATCATCCCTTGCACGCC-3′
	Reversed, 5′-CCTGAGAGGTCACCCAGGTA-3′
TNF-*α*	Forward, 5′-CAGCCGATGGGTTGTACCTT-3′
	Reversed, 5′-ATAGCAAATCGGCTgACGGT-3′
IL-6	Forward, 5′-AGCCAGAGTCCTTCAG AGAGAT-3′
	Reversed, 5′-AGGAGAGCATTGGAAATTGGGG-3′
CAT	Forward, 5′-CACTGACGAGATGGCACACT-3′
	Reversed, 5′-TGTGGAGAATCGAACGGCAA-3′
SOD1	Forward, 5′-CCAGTGCAGGACCTCATTTT-3′
	Reversed, 5′-GTTTACTGCGCAATCCCAAT-3′
SOD2	Forward, 5′-GTAGGGCCTGTCCGATGATG-3′
	Reversed, 5′-CGCTACTGAGAAAGGTGCCA-3′
NQO-1	Forward, 5′-ACTCGGAGAACTTTCAGTACC-3′
	Reversed, 5′-TTGGAGCAAAGTAGAGTGGT-3′
GPX-1	Forward, 5′-GCTCACCCGCTCTTTACC-3′
	Reversed, 5′-GCCGCCTTAGGAGTTGC-3′
NRF1	Forward, 5′-GCACCTTTGGAGAATGTGGT-3′
	Reversed, 5′-GATAAATGCCCGAAGCTGAG-3′
TFAM	Forward, 5′-GCTAAACACCCAGATGCAAAA-3′
	Reversed, 5′-CCTTCTCCATACCCATCAGC-3′

## Abbreviations

ATP: Adenosine triphosphate
CAT: Catalase
COX-2: Cyclooxygenase-2
COX-IV: Mitochondrial complex IV
Dex: Dexamethasone
GAPDH: Glyceraldehyde-3-phosphate dehydrogenase
GPX-1: Glutathione peroxidase-1
HO-1: Heme oxygenase-1
IL-6: Interleukin
LPS: Lipopolysaccharide
mtDNA: Mitochondrial DNA
mtROS: Mitochondrial reactive oxygen species
NO: Nitric oxide
NQO-1: NADP(H) quinone oxidoreductase 1
NRF1: Nuclear factor-erythroid derived 2-related factor 1
NRF2: Nuclear factor-erythroid 2-related factor-2
PCNA: Proliferating cell nuclear antigen
PGC-1*α*: Peroxisome proliferator-activated receptor gamma coactivator 1-alpha
ROS: Reactive oxygen species
SOD1: Cu, Zn-superoxide dismutase
SOD2: Mn-superoxide dismutase
TSG: 2,3,5,4′-Tetrahydroxystilbene-2-O-*β*-D-glucoside
TFAM: Mitochondrial transcription factor A
TNF-*α*: Tumor necrosis factor-*α*
ZnPP: Zinc Protoporphyrin.
